# LC-MS/MS Determination of Modified Nucleosides in The Urine of Parkinson’s Disease and Parkinsonian Syndromes Patients

**DOI:** 10.3390/molecules25214959

**Published:** 2020-10-27

**Authors:** Paulina Gątarek, Joanna Kałużna-Czaplińska, Małgorzata Pawełczyk, Karol Jastrzębski, Joanna Giebułtowicz, Andrzej Głąbiński, Barbara Bobrowska-Korczak

**Affiliations:** 1 Faculty of Chemistry, Institute of General and Ecological Chemistry, Lodz University of Technology, 116 Zeromskiego Street, 90-924 Lodz, Poland; gatarekpaulina@gmail.com (P.G.); joanna.kaluzna-czaplinska@p.lodz.pl (J.K.-C.); 2 Department of Neurology and Stroke, Medical University of Lodz, 90-549 Lodz, Poland; malgorzata.pawelczyk@umed.lodz.pl (M.P.); karol.jastrzebski@umed.lodz.pl (K.J.); andrzej.glabinski@umed.lodz.pl (A.G.); 3 Department of Bioanalysis and Drug Analysis, Faculty of Pharmacy with the Laboratory Medicine Division, Medical University of Warsaw, Poland Banacha 1, 02-097 Warsaw, Poland; jgiebultowicz@wum.edu.pl; 4 Department of Bromatology, Medical University of Warsaw, Faculty of Pharmacy with the Laboratory Medicine Division, Poland Banacha 1, 02-097 Warsaw, Poland

**Keywords:** Parkinson’s disease, PD, parkinsonian syndromes, methylation, modified nucleosides, purine metabolism, chromatographic techniques

## Abstract

Epigenetic modifications play a key role in gene regulation and expression and are involved in numerous cellular processes. Due to the limited research on nucleosides in Parkinson’s disease (PD), it is very important to consider epigenetic factors and their role in the development of PD. The aim of this study was to investigate and compare the levels of modified nucleosides, such as *O*-methylguanosine, *N*^6^-methyl-2′-deoxyadenosine, 1-methyladenosine, 1-methylguanine, 7-methylguanine, 3-methyladenine and 7-methylguanosine in the urine of Parkinson’s disease (PD) patients and the control group, and to verify that the results obtained differ in a subgroup of patients with parkinsonian syndromes. The study group comprised 18 patients with diagnosed idiopathic Parkinson’s disease and four parkinsonian syndromes. The control group consisted of 30 age- and sex-matched neurological patients without confirmation by neuroimaging brain damage and extrapyramidal symptoms. The levels of nucleosides were determined by validated liquid chromatography coupled with the mass spectrometry (LC-MS/MS) method using the multiple reaction monitoring (MRM) mode. Lower levels of *O*-methylguanosine, 3-methyladenine, 1-methylguanine, *N*^6^-methyl-2′-deoxyadenosine and a higher level of 7-methylguanine in the urine of 22 PD patients were observed. Moreover, elevated levels of 1-methyladenosine, 7-methylguanine, and *O*-methylguanosine were observed in the parkinsonian syndrome subgroup. These preliminary results may indicate that modified nucleosides describe metabolic disturbances in the metabolism of purine, which was the most severely affected pathway that mediated the detrimental effects of neuroinflammation on PD.

## 1. Introduction

Parkinson’s disease (PD) is the second most common neurodegenerative disease, after Alzheimer’s disease. The exact mechanisms underlying PD are not clear. There are numerous theories about the specific causes of PD, but they have not yet been proved. The impact of environmental and genetic factors is considered. The disease is more commonly found in people over 50 years of age, but it is also observed in younger patients. Motor symptoms, one of the most characteristic features of the disease, are caused by the loss of dopaminergic neurons in the extrapyramidal system of the brain. This neurotransmitter plays a key role in the control of motor activities [1]. The mechanism that triggers the neurodegenerative process is still unknown. Moreover, there is no information about biological processes which are altered in PD and therefore may cause or may be the consequence of PD, such as inflammation, mitochondrial dysfunction, oxidative stress, protein aggregation and altered Ca^2+^ homeostasis [2]. Idiopathic or sporadic forms of PD with an unknown cause have been described in around 90% of PD patients. In the case of pathogenic mutations, they have been observed in only 10% of patients in monogenic or familial forms of PD [3,4,5,6]. Because of this, PD is considered a complex or prototypic multifactorial disorder [2]. Parkinsonian syndromes may display some clinical features of PD, including bradykinesia and rigidity. They are pathologically heterogeneous, accompanied by degeneration of the nigrostriatal dopaminergic system with neuronal loss and reactive gliosis in the substantia nigra (abnormalities in the presynaptic protein α-synuclein or the microtubule binding protein tau). Parkinsonian syndromes are caused by unknown neurodegenerative processes, genetic factors, toxins, metabolic disturbances or drugs [7,8,9]. One of them is progressive supranuclear palsy (PSP): a neurodegenerative disorder associated with neuronal inclusions composed by tau protein. In PSP, clinical features include other neurologic features not clearly related to parkinsonism, such as dementia and eye movement disorder. This suggests the involvement of brain regions beyond the dopaminergic neurons of the nigrostriatal pathway [9].

Increasing attention is being paid to the role of nucleosides, especially in the regulation of cellular function. Moreover, they also affect the regulation of brain function, may participate in the regulation of cognition memory, sleep and the suppression of seizures. Also, these compounds have been suggested as playing a role in the pathophysiology of neuropsychiatric and neurodegenerative diseases [10,11]. In disorders such as PD, Alzheimer’s disease (AD) and schizophrenia, the levels of nucleosides change extremely indicating their participation in the pathophysiology of the disease [12]. Numerous factors have been examined to determine the effect on the levels of nucleosides in the urine. In healthy subjects, the levels of urinary nucleosides are relatively constant. A little variation was observed between different subjects. Normal nucleosides undergo reutilization or degradation to uric acid, β-alanine and β-aminoisobutyrate, but modified nucleosides, including methylated nucleosides, are excreted intact in urine [13].

It is well known that modified nucleosides are degradation products of nucleic acids and this process is exacerbated in the presence of processes associated with neurodegeneration, carcinogenesis and cell ageing. Nucleosides and nucleobases oxidatively modified are removed from DNA by repair systems. Moreover, they are excreted without further metabolism in the urine. For this reason, the measurement of urinary excretion of these lesions can reveal DNA damage and/or oxidative stress on the whole body. One of the important regulators of cell functions is epigenetic DNA/RNA modifications that directly affect the levels of gene expression. Currently, there is a growing interest in exploring the role of epigenetic changes in different diseases, especially in PD [14,15,16]. These dynamic processes change depending on time and the environment. Epigenetic changes are reversible and heritable modifications in phenotype without alteration of the primary nucleotide sequence [2,4,17]. Epigenetic modifications, including DNA methylation, RNA-mediated processes, and histone modification, play important roles in neural development. Methylation influenced by environmental factors, including hormones, stress, drugs, diet or exposure to environmental chemicals, suggests that these factors can affect DNA methylation [14,15,16]. DNA methylation has been implicated in a diverse range of cellular functions and pathologies [2,4,17]. Urinary excretion of the methylated nucleosides is an indicator of degradation of the methylated RNA, particularly the transfer of ribonucleic acid (tRNA) [14,15,16]. In pathological processes, the RNA turnover is faster than under normal conditions. Under pathophysiological conditions, modified nucleosides are produced. Disruption of normal biological functions may occur through changes in the association/dissociation of specific proteins with methylated bases. In urine, the abnormal concentration of methylated nucleoside may be related to the higher methyltransferase activity and increased RNA turnover. Due to its role in chemical carcinogenesis, increased levels of urinary modified nucleosides were observed in patients suffering from different types of cancers such as colon, rectal, lung, breast and leukemia [16,17,18,19,20,21]. Diseases accompanied by metabolic disorders have characteristic effects on the activity of modifying enzymes, RNA/DNA modifications, and the cell turnover rate. They cause changes in excretion of modified nucleosides and biochemically related compounds [22].

**Figure 1:**
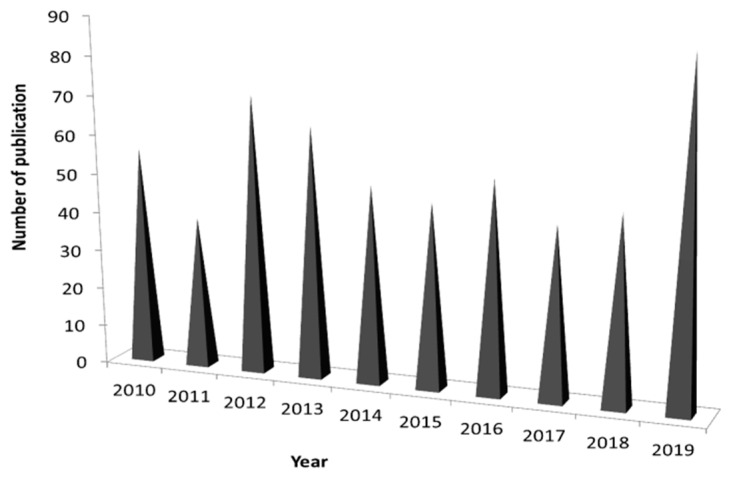
The diagram of frequency of scientific reports on the use of chromatographic techniques in the study of Parkinson’s disease (PD) in 2010–2019. The literature review was based on Sci-Finder sources, sorted by best match for the phrase: chromatographic techniques and Parkinson’s disease or PD.

Epigenetic processes regulate many cognitive and neurobiological functions [23]. DNA methylation is dynamic in PD and changes with the progression of the disease [24]. Recently, epigenetic modifiers have been identified as a potential mediator of environmental factors participating in the pathogenesis of PD. In the literature review, there is a lot of evidence of the application of chromatographic techniques to the analysis of metabolism of PD patients. The recent increase in the number of published studies in this field indicates interest in the chromatographic techniques and Parkinson’s disease (Figure 1).

Saiki et al. (2017) identified nine significant, different levels of concentration of compounds in the serum of PD participants (*n* = 254) compared with controls (*n* = 77) using capillary electrophoresis/liquid chromatography mass-spectrometry (CE-LC/MS). They found nine indicators which may be potential disease severity-independent diagnostic biomarkers for PD, such as 3-methoxytyrosine, urea, homovanillic acid, guanidinosuccinic acid, cortisone, oleoylethanolamine, palmitoylethanolamine, citric acid and deoxycholic acid, which differentiate between PD patients and the control group [25]. Amino acid metabolism in PD was also studied by liquid chromatography-mass spectrometry (LC-MS). LC-MS-based metabolomic analysis in PD is presented in the work of Havelund et al. (2017). They found significantly higher levels of 3-hydroxykynuerine and kynurenic acid in the plasma and lower levels of anthranilic acid both in the plasma and serum of PD subjects. Moreover, they suggested that the 3-hydroxykynurenine/kynurenic acid ratio in the plasma may serve as a biomarker for l-DOPA-induced dyskinesia [26]. Higher levels of acylcarnitine and lower levels of 1-methylhistamine were observed in PD participants by Burté et al. (2017). Their studies suggest that fatty acid oxidation may be used as a diagnostic biomarker for the disease onset and mild cognitive impairment [27]. LC-MS was also used in the research conducted by Roede et al. (2013). They observed a higher level of *N*^8^-acetylspermidine in PD and suggested that altered polyamine metabolism may be used as a predictive marker of rapidly progressing PD [28]. Wuolikainen et al. (2016) investigated plasma and cerebrospinal fluid (CSF) samples of PD (*n* = 22) and the control group (*n* = 28) using gas chromatography (GC) and liquid chromatography (LC)-mass spectrometry (MS) [29]. Sankowski et al. (2020) measured the concentrations of uremic toxins: indoxyl sulfate (IS), p-cresol sulfate (pCS), symmetric dimethylarginine (SDMA), asymmetric dimethylarginine (ADMA), and trimethylamine *N*-oxide (TMAO) in plasma and CSF. In the PD group, they observed a higher level of pCS concentration in CSF, and a higher level of TMAO in plasma, and also a higher level of uremic toxins in CSF in patients with motor fluctuations [30].

Despite numerous studies, the causes of PD are still unknown and further research is needed. Due to the lack of research on nucleotides, and more importantly considering the indications that epigenetics can play a significant role in the development of PD, the main aim of the study was to find out whether there are differences between the levels of *O*-methylguanosine, *N*^6^-methyl-2′-deoxyadenosine, 1-methyladenosine, 1-methylguanine, 7-methylguanine, 3-methyladenine and 7-methylguanosine in the urine of both PD patients and the control group, as well as to verify that the results obtained differ in the subgroup of patients with the parkinsonian syndromes. To the best of our knowledge, these seven modified nucleosides were not determined in the urine of PD patients.

## 2. Results

In the present study, 52 urine samples were analysed. The levels of nucleosides were determined by validated liquid chromatography coupled with the mass spectrometry (LC-MS/MS) method using the multiple reaction monitoring (MRM) mode. The clinical diagnosis differentiating between PD and parkinsonian syndromes was established according to the Movement Disorder Society Clinical Diagnostic Criteria for Parkinson’s disease (MDS-PD criteria) taking into account medical history, neurological examination and the results of additional neuroimaging tests [31]. In accordance with the MDS-PD criteria [31] parkinsonian syndromes are defined based on the three clearly demonstrable cardinal motor manifestations such as bradykinesia in combination with rest tremor, or rigidity, or with both. When a patient is diagnosed with parkinsonian syndromes, the diagnostic criteria for PD are used to determine whether the patient meets criteria for PD as the cause of the parkinsonian syndromes [31]. The clinical condition of the patients was assessed using the Hoehn-Yahr Scale [32]. Patients and controls did not differ significantly from each other with respect to age and gender. Stratification of the tested population, demographic and clinical information are shown in Table 1.

**Figure 2:**
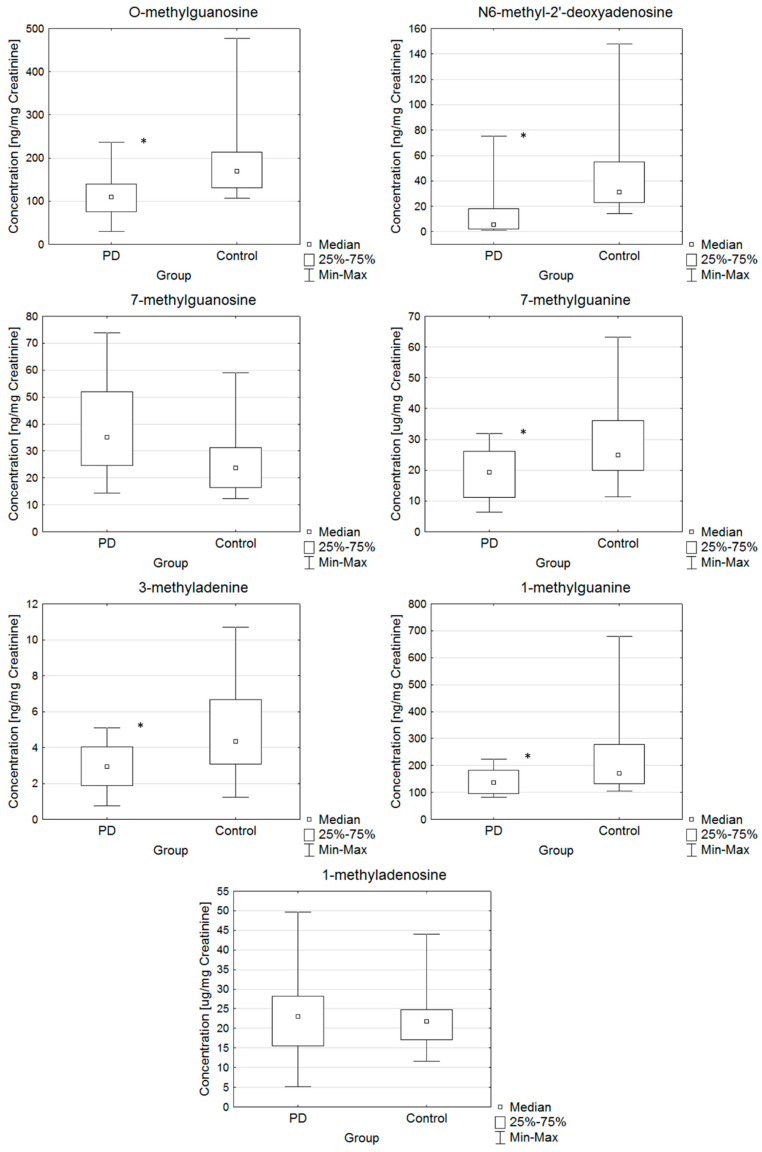
Box and Whisker plots for the compounds determined in the following groups: PD and control. In these box plots, medians (not means) inside the 25–75% interquartile range (IQR) are presented. *—statistically significant with *p*-value higher than 0.05.

The laboratory findings of PD patients and controls are summarized in Table 2 and Figure 2. Table 2 shows the values found for urinary modified nucleosides in the study populations. The results were calculated as a ratio of the analyte of interest and urinary creatinine concentration in the unit ng/mg creatinine (µg/mg creatinine).

Individual differences in the concentrations of *O*-methylguanosine, *N*^6^-methyl-2′-deoxyadenosine, 1-methylguanine, 7-methylguanine and 3-methyladenine between the two groups (PD and control) were found. The obtained results were characterized by high variability. In most subjects, the concentration of *N*^6^-methyl-2′-deoxyadenosine was below the lower limit of quantitation (LLOQ). Application of the Shapiro–Wilk test showed the hypothesis that if the data was normally distributed it could be rejected (*p* < 0.05) for *O*-methylguanosine, 3-methyladenine, 1-methylguanine, *N*^6^-methyl-2′-deoxyadenosine, 7-methylguanine and 7-methylguanosine, but not by 1-methyladenosine (*p* = 0.0579). The levels of modified nucleosides were compared between the two groups using Student’s t-test and Mann-Whitney U test, as shown in Table 2. Individual differences in the levels of modified nucleosides between the two groups (PD and control) were found after performing the Mann-Whitney U test. The application of the Mann-Whitney U test showed a significant difference between the examined groups. The differences with *p*-value lower than 0.05 were considered significant. The application of the Mann-Whitney U test showed a difference in the level of *O*-methylguanosine, 3-methyladenine, 1-methylguanine, *N*^6^-methyl-2′-deoxyadenosine and 7-methylguanine (*p* < 0.05). For 1-methyladenosine and 7-methylguanosine, no statistically significant difference was observed between the PD and control groups. Patients were found to have lower mean and median urine levels of *O*-methylguanosine, 3-methyladenine, 1-methylguanine, 7-methylguanine and *N*^6^-methyl-2′-deoxyadenosine compared to the control group (*p* < 0.05). Based on the median difference, the average difference in the levels of urinary nucleosides between the PD and control groups was 59.7 ng/mg creatinine, 1.4 ng/mg creatinine, 34.7 ng/mg creatinine, 25.7 ng/mg creatinine, respectively for *O*-methylguanosine, 3-methyladenine, 1-methylguanine and *N*^6^-methyl-2′-deoxyadenosine.

Individual differences in the levels of nucleosides between the two groups (PD and control) were found (Figure 2). Box and Whisker plots allow simultaneous evaluation of the differences and variation of the results in the content of modified nucleosides determined by the study group.

**Figure 3:**
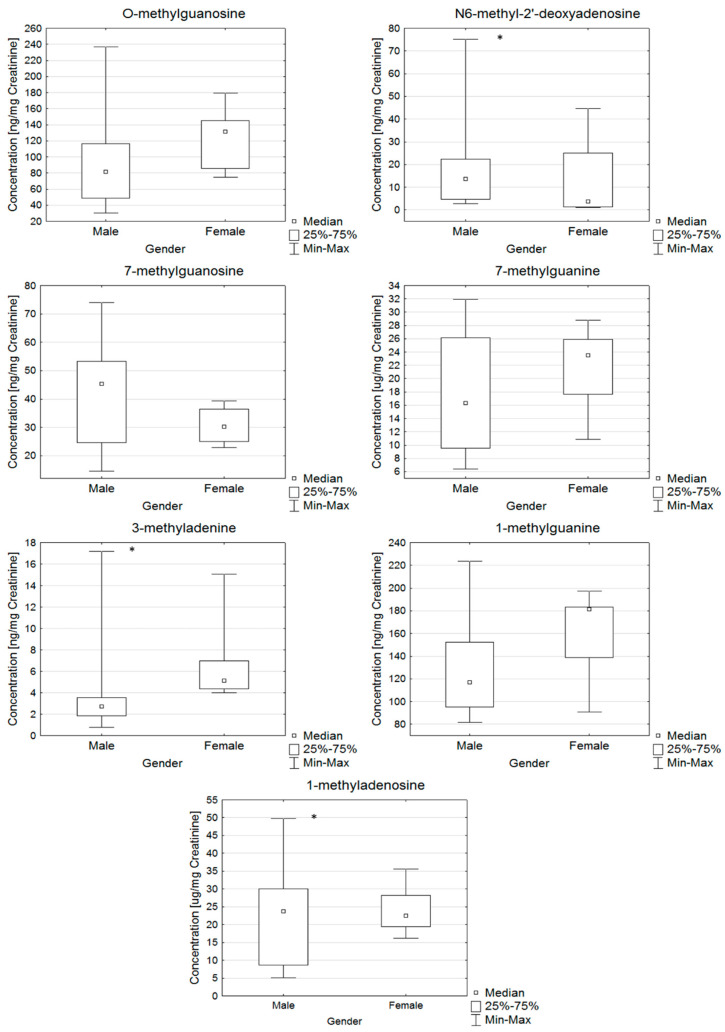
Box and Whisker plots for the compounds determined in the PD group gender-categorized. In these box plots, medians (not means) inside the 25–75% interquartile range (IQR) are presented. *—statistically significant with *p*-value higher than 0.05.

Additionally, individual differences in the levels of nucleosides for a group of participants with PD based on gender (Figure 3) were found. Statistically significantly higher levels of 1-methyladenosine, 3-methyladenine and 7-methylguanosine were observed in women than in men (Figure 3).

Individual differences in the levels of nucleosides for subgroups of participants with PD, parkinsonian syndromes and the control group were found. Elevated levels of 1-methyladenosine, 7-methylguanine and *O*-methylguanosine were observed in the subgroup with parkinsonian syndromes compared to the group of people diagnosed with PD and the control group. Due to the small number of participants, further studies are required. Moreover, a correlation analysis was performed for the examined compounds. The statistical analysis showed a positive correlation between *O*-methylguanosine and 7-methylguanine (correlation value PD = 0.55, control = 0.72). In the PD group, we found a stronger positive correlation between the concentration of *O*-methylguanosine and 1-methyladenosine (correlation value 0.82) and a weaker correlation between 1-methyladenosine and 7-methylguanine(correlation value 0.68).

**Figure 4:**
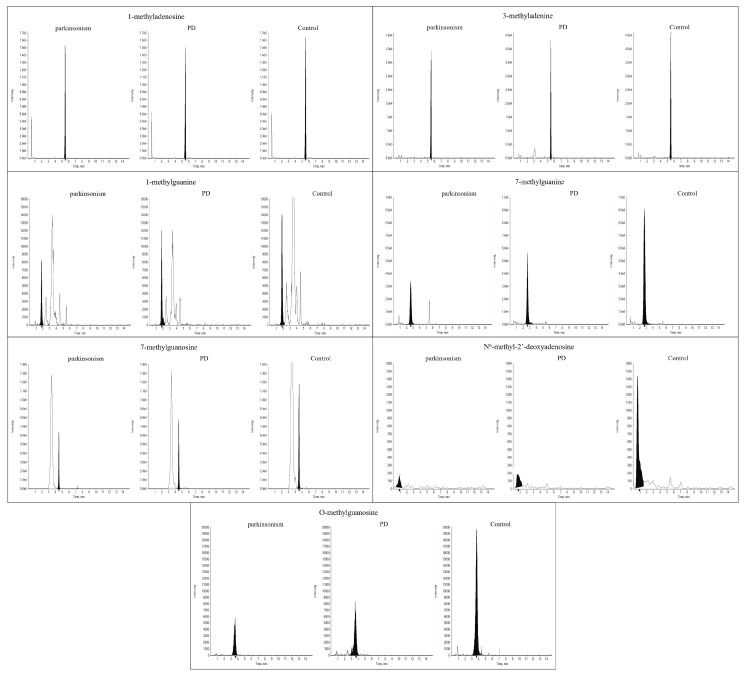
Typical extracted chromatograms of the analytes collected from control, PD and parkinsonian syndromes subgroups.

Typical extracted chromatograms of the analytes in the urine samples collected from PD and control participants are presented in Figure 4.

## 3. Discussion

In the literature, the epigenetic factors which can be associated with the increase in neurodegenerative disease are described. Some studies suggested that DNA/RNA oxidation is involved in neurodegeneration and enhances neuroinflammation mechanisms. The major pathological mechanisms in PD are loss of dopaminergic neurons in the *substantia nigra* and a confluence of mitochondrial dysfunction, oxidative stress, microglia activation and synucleinopathy, which contribute to neural cell death. Our results showed an alteration in the urine levels of *O*-methylguanosine, *N*^6^-methyl-2′-deoxyadenosine, 1-methylguanine, 7-methylguanine and 3-methyladenine of PD patients and proved that the purine metabolism in PD is different from the purine metabolism in control groups. Purine metabolism was the most significantly affected pathway which mediated the detrimental effects of neuroinflammation on PD. Other research indicated a relationship between the altered purine metabolism and the pathology of PD. In the metabolic profile of people with PD, scientists observed altered levels of adenosine, inosine, guanosine, hypoxanthine and uric acid, which suggested that neuroinflammation should induce a significant decrement in adenosine and high levels of catabolic intermediates. Moreover, the level of adenosine was correlated with the severity of parkinsonian symptoms. Adenosine metabolism and the adenosinergic pathway can be promising therapeutic targets for the treatment of PD [33].

Several studies have confirmed that 1-methyladenosine, 1-methylguanosine and 8-hydroxydeoxyguanosine (8-OHdG) are important for modifying the RNA molecule functions which regulate and control protein translation and gene transcription. One of the modified nucleosides, 1-methyladenosine, has been used as an indicator of the tumor burden. This urinary compound is biosynthesized at a post-transcriptional level in tRNA. In normal human urine, it is present at the level of approximately 2.2 mg in 24-h collection [34]. Adenosine methylation in a subset of mRNAs is important for the dopaminergic signaling pathway which is related to the pathogenesis of PD. The most prevalent internal modification occurring in the mRNA of eukaryotes is N^6^-methyladenosine. This compound plays an important role in the post-transcriptional regulation. In the literature, a lot of information about the biological significance of *N*^6^-methyladenosine modification in the nervous system is presented. Moreover, dysregulation of *N*^6^-methyladenosine has been shown to be related to degenerative and neurodevelopmental diseases [35].

The lack of conclusive evidence that PD is only a genetic or environmental disorder implies that epigenetic factors may have an impact on susceptibility to PD. In normal and pathogenic brain development, a critical gene expression regulatory mechanism is DNA methylation. An imbalance between exposure to free radicals or other reactive species and antioxidant defenses is characteristic of oxidative stress. Moreover, oxidative stress has been implicated in the pathogenesis of different neurodegenerative diseases, including PD and AD [36,37,38]. These may be related to changes in protein clearance mechanisms and mitochondrial function. The redox imbalance in PD causes oxidative damage to DNA which increases with the progression of the disease [39,40]. Compounds such as 3-methyladenine and 7-methyladenine are considered to be markers of DNA damage from exposure to methylating agents. Bolner et al. (2018) suggested a decrease in urinary 2-deoxy-guanosine (2-dG) with the PD progression [41]. 8-OHdG is one of the most abundant oxidative DNA base damage products. As it is well known, urine 8-OHdG is considered a marker of oxidative DNA damage and is frequently used as a marker for susceptibility to cancer [42] and neurodegenerative diseases [43]. The concentration of 8-OHdG in the urine and plasma depends on the efficacy of the DNA repairing system and its oxidation rate [44]. Research conducted by Bolner (2011) indicated that 8-OHdG and the ratio 8-OHdG/2-dG were significantly different in controls and PD patients. In PD plasma samples, the ratio 8-OHdG/2-dG was significantly higher compared to controls [44]. Other studies demonstrated increased 8-OHdG levels in the serum, cerebrospinal fluid (CSF) and urine of PD patients, indicating that 8-OHdG may be a useful biomarker in tracking PD progression due to the fact that 8-OHdG increased with the progression of the disease. Several lines of evidence have shown that oxidative stress contributes to neurodegeneration of PD. Increased lipid peroxidation, reduced glutathione, increased 8-OHdG and decreased antioxidant capacity against hydroxyl radical in *substantia nigra* compacta of PD were reported [45]. Moreover, urinary 8-OHdG levels in PD patients correlated with the degrees of hallucination [46].

In the present study, the liquid chromatography/mass spectrometry method for the evaluation of levels of modified nucleosides in urine samples of PD and the control group was applied. Determination of modified nucleosides in biological fluids may serve as a non-invasive diagnostic method for the disease with metabolic disorders; however, our study has some limitations. The study population was relatively small. Another limitation is the sensitivity and specificity of diagnostic criteria (they are around 80% for a clinically probable PD) [31]. Therefore, further studies are required and our results must be cautiously interpreted. Since modified nucleosides can play a role in many biological functions, it is possible that they may contribute to the pathophysiology of these disorders more than expected. Whatever the impact of nucleosides in other neurological disorders, its role in PD needs further elucidation. At present, there is no clear answer on whether methyl marks of RNA play any roles in PD.

## 4. Materials and Methods

### 4.1. Subjects

The study group (PD) consisted of 22 patients with diagnosed idiopathic Parkinson’s disease (*n* = 18) and parkinsonian syndromes (*n* = 4). The clinical diagnosis differentiating between PD and parkinsonian syndromes was established according to MDS-PD criteria taking into account medical history, neurological examination and the results of additional neuroimaging tests [31]. The clinical condition of the patients was assessed using the Hoehn-Yahr Scale [32]. PD patients were treated using l-dopa (82%, 100–1125 mg/day, mean dose 590 mg/day), amantadine (45%, 100–300 mg/day, mean dose 210 mg/day), and dopamine agonists (41%, 4–8 mg/day, mean dose 6.7 mg/day). None of them obtained monoamine oxidase B inhibitors or catechol-*O*-methyl transferase inhibitors. The control group consisted of 30 age- and sex-matched neurological patients without confirmation by neuroimaging brain damage and extrapyramidal symptoms.

Patients with severe liver disease, renal failure, cancer and chronic inflammatory diseases were not enrolled in the study. The PD and control groups were recruited from the patients hospitalized in the Department of Neurology and Stroke, Medical University of Lodz, Poland. Consent was obtained for experimentation with human subjects. All subjects gave their informed consent for inclusion before they participated in the study. The study was approved by the Bioethics Committee of the Medical University of Lodz, Lodz, Poland (No. RNN/399/17/KE) and the work was conducted in accordance with the Declaration of Helsinki ethical guidelines. All procedures were carried out with the adequate understanding and written consent of the subjects. 

Fifty-two morning urine samples from participants were collected into sterile containers. Morning urine was collected after the night in order to minimize the impact of diet. Urine samples were thoroughly mixed in order to maintain homogeneity and aliquoted. Urine was collected into 1.5 mL Eppendorf tubes and stored at −20 °C until LC-MS analysis.

### 4.2. Sample Preparation

Urine samples were thawed at room temperature. Aliquots of 0.1 mL were mixed with tubercidin (0.1 mL, 1 µg/mL) and acetonitrile (0.6 mL), vortexed at high speed (3 min) and centrifuged (5 min at 10,000 g). Onto the analytical column, 5 µL of supernatant was injected.

### 4.3. Urinary Creatinine Determination

The level of the modified nucleosides and bases in urine was standardized by conversion to the creatinine level. The creatinine level was investigated using a commercially available test Creatinine Liquicolor Jaffe’-Reaction, Human Gesellschaft für Biochemica und Diagnostica mbH, Max-Planck-Ring 21, 65,205 Wiesbaden, Germany.

### 4.4. Analytical Methods

Modified nucleobases and nucleosides were determined by the high performance liquid chromatography coupled to mass spectrometry (LC-MS/MS) method using a multiple reaction monitoring mode on Agilent 1260 Infinity (Agilent Technologies, Santa Clara, CA, US) coupled to QTRAP 4000 (AB Sciex, Framingham, MA, USA) and SeQuant^®^ ZIC^®^-HILIC column (50 mm × 2.1 mm; 5 μm) (Merck, Darmstadt, Germany). The column was maintained at 40 °C at a flow rate of 0.5 mL/min. Gradient elution using 20 mM ammonium acetate as eluent A and acetonitrile with 0.2% formic acid as eluent B was applied. The gradient (%B) was as follows: 0 min, 95%; 1 min, 95%; 7 min, 50%; and 8 min, 50%. The following transitions were used for quantification (*m/z*): 166 > 135 (1-methylguanine), 166 > 79 (7-methylguanine), 298 > 152 (2′-*O*-methylguanosine), 150 > 123 (3-methyladenine), 282 > 55 (1-methyladenosine), 266 > 150 (*N*^6^-methyl-2′-deoxyadenosine) and 267 > 135 (Tubercidin, Internal Standard).

### 4.5. Validation

The method was validated for selectivity, calibration curve, precision, accuracy, stability, matrix effect, dilution integrity and carryover according to the guidelines of the European Medicines Agency (EMA). The results were published previously [47]. The linearity of the calibration curve was evaluated via the R^2^ regression coefficient >0.995. The within run accuracy and precision (all samples were analyzed in one run) ranged from 89% to 108% and from 0.2% to 4.3%, respectively. In this method, the relative matrix effect for all analytes in urine did not exceed 15%, as required by validation criteria. It was found that the quantification of nucleosides performed by LC-MS/MS using tubercidin as an internal standard was of acceptable accuracy and precision [47].

### 4.6. Statistical Analysis

The results are presented as the mean ± standard deviation (SD). All variables were checked for normality by the Shapiro–Wilk’s test. Student’s *t*-test and nonparametric Mann-Whitney U test were used, as appropriate, to compare the concentration of nucleosides between the studied groups. All comparisons used to indicate statistical significance were two-sided with a *p*-value of less than 0.05. Subsequently, the correlation analysis for groups of patients and the controls was carried out to discover possible relations among the variables. Statistical analysis was performed using Statistica 9.0 software (StatSoft, Polska STATISTICA, version 9.0, Quest Software, Aliso Viejo, CA, USA).

## 5. Conclusions

Because purines are the core of DNA, RNA, nucleosides and nucleotides which participate in a wide variety of crucial metabolic pathways, it is very important to examine the changes in the expression of genes encoding enzymes involved in purine metabolism in PD. Epigenetic factors have an impact on susceptibility to PD. The results showed lower levels of urinary nucleosides, such as *O*-methylguanosine, *N*^6^-methyl-2′-deoxyadenosine, 1-methylguanine, 7-methylguanine and 3-methyladenine in PD participants. For 1-methyladenosine and 7-methylguanosine, no statistically significant difference was observed between the study groups. Moreover, individual differences in the levels of nucleosides for subgroups of participants with PD, parkinsonian syndromes and controls were found. Higher levels of 3-methyladenine, 1-methyladenosine and 7-methylguanosine were observed in women than in men. This information could allow a better understanding of the primary regulation of purine-related genes and their possible implications for the pathogenesis of the disease. Because PD is a multifactorial disease, a more precise diagnosis and personalized medication are required in order to obtain an optimal outcome. To better understand PD, the impact of chemical, biological, genetic and other factors on the pathophysiology of the disease needs to be considered. For this purpose, it is necessary to apply analytical methods used for the determination of potential biomarkers of PD patients or patients at risk of developing PD. These methods are a powerful tool for a holistic understanding of the metabolic pathways and also early diagnosis, monitoring and prognosis of the disease progression.

## Figures and Tables

**Table 1:** Stratification of the tested population (mean ± SD).

Study Population	PD	Control
Participants	22	30
Gender		
Female	7	16
Male	15	14
Age (years)	69.7 ± 6.6	63.0 ± 7.2
BMI	26.2 ± 4.1	30.2 ± 3.8
Disease duration (years)	5.7 ± 4.3	-
Hoehn and Yahr scale	2.6 ± 1.1	-
H–Y1	5 (23.8%)	-
H–Y2	4 (19.1%)	
H–Y3	7 (33.3%)	
H–Y4	5 (23.8%)	

**Table 2:** Values obtained for quantification of determined nucleosides in urine samples of PD (*n* = 22) and control adults (*n* = 30).

Name of Nucleoside	Unit	Population	Mean ± SD	Median	Min	Max	LQ	UQ	SE	*p*-Value
*O*-methylguanosine	ng/mg Creatinine	PD	107.3 ± 52.7	109.4	30.1	236.5	75.0	139.4	12.1	0.0002
		Control	187.9 ± 83.1	169.1	107.5	478.0	130.7	213.7	17.7	
3-methyladenine	ng/mg Creatinine	PD	2.9 ± 1.3	2.9	0.8	5.1	1.9	4.0	0.3	0.0280
		Control	5.0 ± 2.9	4.3	1.2	10.7	3.1	6.6	0.6	
1-methylguanine	ng/mg Creatinine	PD	139.7 ± 46.6	134.8	81.6	223.8	95.0	183.5	12.0	0.0257
		Control	228.1 ± 142.8	169.5	104.0	680.0	133.4	277.3	30.4	
*N*^6^-methyl-2′-deoxyadenosine	ng/mg Creatinine	PD	15.8 ± 25.1	5.1	1.0	75.3	2.2	17.9	8.9	0.0034
		Control	48.4 ± 40.1	30.8	14.3	147.8	23.0	54.9	11.1	
1-methyladenosine	μg/mg Creatinine	PD	23.4 ± 12.7	23.0	5.2	49.7	15.5	28.2	2.7	0.7704
		Control	22.5 ± 8.3	21.8	11.6	44.1	17.1	24.7	1.7	
7-methylguanine	μg/mg Creatinine	PD	19.3 ± 8.3	19.3	6.4	31.9	11.2	26.0	2.1	0.0315
		Control	28.0 ± 12.6	24.8	11.5	63.1	20.0	36.2	2.5	
7-methylguanosine	ng/mg Creatinine	PD	38.6 ± 17.6	35.0	14.5	73.9	24.6	51.9	4.9	0.0616
		Control	27.1 ± 14.1	23.6	12.4	59.1	16.3	31.2	3.5	

## Abbreviations

2-dG	2-deoxy-guanosine
8-OHdG	8-hydroxydeoxyguanosine
CE-LC/MS	capillary electrophoresis/liquid chromatography mass-spectrometry
CSF	cerebrospinal fluid
GC	gas chromatography
IQR	interquartile range
LC-MS	liquid chromatography-mass spectrometry
LLOQ	lower limit of quantification
LQ	lower quartile
Max	maximum
Min	minimum
MS	mass spectrometry
PD	Parkinson’s disease
SD	standard deviation
SE	standard error
UQ	upper quartile
